# Safety work and risk management as burdens of treatment in primary care: insights from a focused ethnographic study of patients with multimorbidity

**DOI:** 10.1186/s12875-018-0844-0

**Published:** 2018-09-08

**Authors:** Gavin Daker-White, Rebecca Hays, Thomas Blakeman, Sarah Croke, Benjamin Brown, Aneez Esmail, Peter Bower

**Affiliations:** 1 NIHR Greater Manchester Patient Safety Translational Research Centre (Greater Manchester PSTRC), Division of Population Health, Health Services Research and Primary Care, School of Health Sciences, Faculty of Biology, Medicine and Health, University of Manchester, Manchester Academic Health Science Centre, Manchester, UK; 2 NIHR School for Primary Care Research, Division of Population Health, Health Services Research and Primary Care, School of Health Sciences, Faculty of Biology, Medicine and Health, University of Manchester, Manchester Academic Health Science Centre, Manchester, UK; 3 NIHR Collaboration in Applied Health Research and Care Greater Manchester, Division of Population Health, Health Services Research and Primary Care, School of Health Sciences, Faculty of Biology, Medicine and Health, University of Manchester, Manchester Academic Health Science Centre, Manchester, UK; 4 Division of Nursing Midwifery and Social Work, School of Health Sciences, Faculty of Biology, Medicine and Health, University of Manchester, Manchester Academic Health Science Centre, Manchester, UK; 5 Centre for Health Informatics, School of Health Sciences, Faculty of Biology, Medicine and Health, University of Manchester, Manchester Academic Health Science Centre, Manchester, UK

**Keywords:** Multimorbidity, Patient safety, Primary care, Qualitative study, Risk, Burden of treatment

## Abstract

**Background:**

In primary health care, patient safety failures can arise in service access, doctor-patient relationships, communication between care providers, relational and management continuity, or technical procedures. Through the lens of multimorbidty, and using qualitative ethnographic methods, our study aimed to illuminate safety issues in primary care.

**Methods:**

Data were triangulated from electronic health records (EHRs); observation of primary care consultations; annual interviews with patients, (informal) care providers and GPs. A thematic analysis of observation, interview and field note material sought to describe the patient safety issues encountered and any associated factors or processes. A more detailed longitudinal description of 6 cases was used to contextualise safety issues identified in observation, interviews and EHRs.

**Results:**

Twenty-six patients were recruited. Events which could lead to harm were found in all areas of a framework based on published literature. “Under” and “over” consultation as a precursor of safety failures emerged through thematic analysis of observation and interview material. Other findings concerned workload (for doctors and patients) and the limitations of short consultation times. There were differences in health data collected directly from the patients versus that found in EHRs. Examples included reference to a stroke history and diagnoses for CKD and hypertension. Case study analysis revealed specific issues which appeared contextual to safety concerns, mostly around the management of polypharmacy and patient medication adherence. Clinical imperatives appear around risk management, but the study findings point to a potential conflict with patient expectations around investigation, diagnosis and treatment.

**Discussion:**

Patient safety work involves further burdens on top of existing workload for both clinicians and patients. In this conceptualisation, safety work seemingly forms part of a negative feedback loop with patient safety itself. A line of argument drawn from the triangulation of findings from different sources, points to a tension between the desirability of a minimally disruptive medicine versus safety risks possibly associated with ‘under’ or ‘over’ consultation. Multimorbidity acts as a magnifier of tensions in the delivery of health services and quality care in general practice. More attention should be put on system design than patient or professional behaviour.

## Background

The patient safety literature traditionally draws on incidents [1] such as adverse events [2], which have “root causes” embedded in the safety culture of an organisation [2, 3]. In a synthesis of the literature on patient safety in primary care, a distinction was drawn between “preventable adverse events” (such as “incorrect drug administration”) and “process errors” (such as errors in clinical judgement) [4]. However, there are a variety of definitions of medical “error” and the nature of uncertain outcomes in primary care means an error may sometimes only become visible with the benefit of hindsight [5]. Further, “everyday” errors (such as missing test results) may not be conceived of as errors at all [5].

In primary care the elements of safety are as much embedded in communication issues as they are in the technological or administrative aspects of care [6]. However, a qualitative study of US-based family physicians found that most medical errors “arose from healthcare systems dysfunction” [7]. From the patient perspective, “safety” appears as a subjective feeling that is “fluid” and “negotiated” according to ongoing interactions with health services [8]. In this context, patients with clinical multimorbidity [9] are of interest as they are more likely to have a large number of interactions with medical services [10] and prescribed medicines, putting them at increased risk of safety failures [11].

Thus, safety in primary care appears as a potentially nebulous concept [12] although the relevant factors involved can be attributed to patients, doctors and healthcare systems [13]. From our perspective safety incorporates a range of processes or events and can include, variously, serious adverse events, medical errors (e.g. in diagnosis or prescription), technical problems (e.g. failure to access the medical record) and issues in communication and inter-personal relationships involving a patient and the carers around them [13]. Through a lens focused on both doctor and patient, we were interested to see whether we could identify discernible failures and link these to precursor events or the surrounding clinical and social context.

In order to explore patient safety in primary care, we selected to study patients with multimorbidity *(*“the co-existence of two or more chronic conditions, where one is not necessarily more central than the others”), as they are known to be at increased risk of poor care outcomes [14]. Broadly, the ultimate objective of the wider study was to identify areas in which patients could become more active agents in their own safety management [15]. We have previously reported baseline findings which showed that patients appeared to feel that their safety was threatened “when they felt their needs were ignored, or when they perceived responses as inappropriate or insensitive” [16].

Adverse consequences for patients with multimorbidity may arise in relation to care continuity [17, 18], care transitions [16] and polypharmacy [19]. Complexities of care can create self-management challenges for patients [20] including “treatment burdens” [21], such as dealing with polypharmacy [21] and interpreting sometimes contradictory advice received from different professionals [22]. The consequences of multimorbidity in primary care are influenced by contemporary workloads for general practitioners (GPs), which are seen as “unsustainable” [23]. A report by the UK King’s Fund, highlighted the additional workload challenges posed by the rising numbers of people living with multimorbidity [24]. It has been argued that consultation times should be “substantially enhanced” beyond 9.2 min in consequence [24].

Patients with multimorbidity complicate clinical decision-making [25, 26]. A qualitative study of primary care health professionals’ views on these issues found a tendency to deal with patients’ needs in order of priority “until consultation resources were exhausted, when further management was deferred” [27]. A study of Dutch GPs’ views on the clinical management of multimorbidity found that a “personal doctor-patient relationship was considered a major facilitator” whereas “presence of mental health problems was regarded as a complicating factor” [28]. A review and synthesis of related studies pointed to GPs feeling isolated in relation to clinical decision-making and a perceived need for “bidirectional communication” between care providers [29]. Similar issues were evident in a recent Scandinavian study [30]. National Institute for Health and Care Excellence (NICE) guidelines on the clinical management of multimorbidity recommended better co-ordination and tailoring of patient care [31].

“Treatment burden” in multimorbidity has received increasing attention. Gallacher et al. describe the work faced by patients which coalesces around care continuity, communication, access to services, polypharmacy and “fragmented and poorly organized care” [32]. The consequences of treatment burden include poor health outcomes, lack of adherence to prescribed treatments, “avoidable resource use” and a consequential burden for informal carers [33]. Treatment burden varies according to an individual’s ability to cope [34] and the personal or social resources available, including “resilience” [35]. May et al. make clear that a patient’s “relational networks will often include healthcare and other professionals” [36] although subsequent studies have tended to focus on treatment burden for patients, sometimes with the aim of informing a “minimally disruptive medicine” [37] or in efforts to mobilise patient resources to access health services and enact self-care [38]. A meta-analysis of “active” patient safety incidents (i.e. adverse events) and their precursors (e.g. prescription or diagnostic errors) concluded that “multimorbidity involving mental health may be a key driver of safety incidents” [39].

To date, studies into multimorbidity and safety have focused on polypharmacy [40, 41]. In a thematic analysis of primary care patient safety incident reports in older adults, most unsafe care was related to dispensing and prescription errors, failures in the transfer of information between sites of care and errors in clinical decision-making [42]. Although such errors are important, a meta-synthesis of published studies pointed to patient safety in primary care as a subjective feeling or moral framework as opposed to more tangible or recordable medical errors or adverse events [13]. With notable exceptions [43], previous qualitative studies of patient safety in primary care have tended to adopt a cross-sectional approach and limit data collection methods to individual or group interviews [13]. Although useful, such studies may lack a detailed assessment of the wider context in which problems occur, and cannot explore how problems develop and are managed over time.

The aims of the study were to describe the safety issues identified in a cohort of primary care patients with multimorbidity; and, to explore the clinical and social context in which patient safety concerns arise and play out. These objectives were addressed via a longitudinal, multi-method qualitative study in the form of a focused ethnography [44]. The principle aim of the analysis was to construct a line of argument concerning the circumstances under which patient agency in safety monitoring might be bolstered.

## Methods

The study methods are detailed elsewhere [15]. Twenty-six patients were purposefully recruited following identification by participating GPs in Greater Manchester and enrolled for 24 months. Attempts were made to interview patients face-to-face every 12 months and after primary care consultations. On occasion, informal carers were also present at these interviews or were interviewed separately. Patient’s GPs and/or practice nurses were interviewed at year 1 and year 2. We aimed to observe at least two primary care consultations each year with each participant. Some participating patients completed a health care diary. Data were extracted from practice electronic health records for each participant at study months 0,12 and 24. Where possible, interviews and observations were audio recorded; with informed consent to use a recorder verbalised at the start of each interview or observation by each participant present. Where one or more participants did not consent to the use of an audio recorder, notes were taken by hand. The fieldworkers (RH, GD-W SC), who were experienced qualitative health researchers, recorded their own impressions and observations as field notes. During the course of the study, one patient dropped out (data excluded) and three died (data retained).

For the purposes of the thematic analysis of interview, observation and diary material, all data were read several times by the first author and selected data which fit our a priori framework [15] was extracted and further organised and analysed by the wider study team with the aid of Quirkos software (www.quirkos.com). The framework was based on the findings of three previous qualitative studies of patient safety in primary care [45–47]. We coded inductively from a subset of interview and observation material (from all participants) that was directly pertinent to patient safety as it fit a taxonomy based on these international studies. We included any surrounding context. We have not shown our working here, as referees pointed out that this data does not add anything to what is already known in the literature. These situations and events could also be used to more easily identify patient and GP participants.

For the case study [48] analysis, we began by comparing information on medical conditions, prescribed medicines and allergies or drug sensitivities as described by patients or found in EHRs. The primary purpose of this exercise was not to discern whether “errors of fact” lay either with the patient or with the system, but rather to identify cases where more detailed analysis could provide context around identified patient safety concerns. A deviant case [49] – where there were no discrepancies between information divulged by patient and practice – was also selected – for comparison, although our analytical approach is primarily descriptive.

For the six cases identified, we analysed all of the data collected for each individual, i.e. EHRs, paper prescriptions (where available), transcripts and field notes from participant interviews and observations of clinical consultations. All data were anonymised at, or immediately following data collection and all participant identifiers are pseudonyms. Ethical approval was obtained. Informed consent was obtained from all participant GPs, patients and carers. Ongoing assent was verbalised in subsequent interviews or observation sessions. Certain unique clinical features of individual cases have been redacted or blurred (e.g. described in less specific terms) and the original datasets are not shown in order to protect participant anonymity.

## Results I – Health records analysis

Of 26 patients recruited at baseline (Table 1), 3 died during the course of the study and one withdrew following concerns that the study would saddle them with additional burden. Comparing information on active health conditions, prescribed medicines and allergies or drug sensitivities found discrepancies for all patient participants, bar one. Thirteen (52% of 25) patients did not mention drug allergies or sensitivities as recorded in their EHR and there were 4 instances where drug allergies mentioned by the patient were not found in the EHR. Sixteen (64% of 25) participating patients did not mention some of the active health conditions recorded in the EHR. However, this was perhaps to be expected in a group of people who had been deliberately selected as having multiple health problems. It was also unsurprising that 4 elderly male participants chose not to disclose to a younger, female researcher that they had erectile dysfunction problems or were in receipt of sildenafil prescriptions.

**Table 1:** Participant characteristics. Demographics of patient participants (from patient reported data at baseline)

		N	%
Gender	Female	14	53.85
	Male	11	42.31
Age group	65–74	10	38.46
	75+	15	57.69
Prescribed medicines	≤6	7	26.92
	7–10	7	26.92
	11+	11	42.31
Long-term conditions	2–3	7	26.92
	4–5	7	26.92
	6+	11	42.31
Requires mobility aid*	Yes	9	34.62
	No	16	61.54
Living status	Alone	7	26.92
	With partner	15	57.69
	With family member	3	11.54
Index of Multiple Deprivation (IMD) Decile**	1–2	9	34.62
	3–4	13	50.00
	5+	3	11.54
Total number of patients		25	100.00

*That is a crutch, stick, walker or wheelchair

**Where 1 is most deprived 10% of Lower Layer Super Output Areas

(data from http://imd-by-postcode.opendatacommunities.org/)

There were more surprising omissions; principally the participant who appeared not to know that they had previously had a TIA or stroke. In another case, a suspected TIA was mentioned by the patient but not seen in the EHR. Two patients did not mention a diagnosis of hypertension. Three did not mention a CKD diagnosis, although this could have been asymptomatic in the two participants with CKD Stage 3A. In three cases, it appeared as though some of the patient’s principal health concerns were not recorded in the EHR as they did not have a definitive diagnostic label or could not be treated effectively, as in a woman with recurrent skin infections, another with “age spots” and a man with a “lazy eye.”

On the basis of the above exercise, 6 cases were selected for more detailed consideration as they seemed to highlight potential areas where safety issues would arise or were seen to have arisen. Kathleen was seen at an Urgent Care Centre following a fall in year one and the GP had put a Personalised Care Plan in place which included “admissions avoidance care.” Pamela’s son was concerned about her medication compliance and the GP suspected cognitive impairment. Deborah was selected primarily because she told the researcher about memory changes which she had withheld from her GP. However, from the GP’s perspective, the main safety considerations in her care related to the possible risks of hospital admission resulting from specialised medication that she was taking. Alan was admitted to hospital after collapsing at an event in year 2. This, along with further episodes of “feeling faint,” he associated with a statin prescription (although “feeling faint” is not a reported side effect of statins [50]) which was subsequently stopped. Helen was taking more prescribed medicines than any other case considered in detail, and as with Deborah, there were concerns about the possibility of “stomach bleeding or kidney problems” and “ending up in hospital” as a result. Finally, Victoria was selected as she was the only participating patient where no discrepancies were found between medical information collected from herself versus that found in the EHR. She only had three active health conditions, including heart failure and diverticular disease. Further description of each of these cases is considered below.

Comparison of numbers of primary care consultations for the included cases during the 2 year study period mainly underlined the low number of primary care appointments seen in Victoria (the “deviant case”) when compared with others. Victoria only had 6 consultations in 24 months (data missing for one month) whereas the remaining participant cases were usually seen in primary care at least once a month. Deborah seemed to have a more overall pattern of regular and consistent use when compared with the others, although she had 3 consultations in month 20. The remaining cases revealed episodes involving periods of large numbers of consultations. Thus, Pamela had 12 consultations in 4 months (months 8–11), Alan had 6 consultations in one month (month 19) and Helen had 23 consultations during months 16–22 inclusive (an average of 3 per month).

Across the study period, participating patients were being prescribed between 7 and 18 items in primary care. Victoria, the “control” was in the lowest position in this regard. Data on secondary care appointments showed that Victoria and Pamela were the only participants not seen in secondary or specialist care for the duration of the study. Alan had only one incident involving secondary care – an emergency admission. Three participants: Kathleen, Deborah and Helen were usually visiting hospitals or specialist clinics every month and each of them had two emergency or urgent care admissions during the course of the study. Combining data on total primary and secondary care appointments over the 24 months of data collection shows that Victoria, the control, had a total of 8 appointments, whereas Kathleen, Pamela and Deborah had between 59 and 67. Helen was also likely in this latter category (with a total of 52 appointments), although data on her secondary care appointments was missing for 5 straight months. On the basis of the included figures, these latter four participants had a mean of 60 total health appointments, or 2.5 per calendar month.

## Results II - Case studies

### Kathleen

In month 9 of the study Kathleen had a fall and was seen at an urgent care centre. Following this incident, her GP instigated a Personalised Care Plan, including “admission avoidance care.” In month 16, Kathleen presented at the surgery feeling “dizzy and wobbly” with “lots” of pain in her back. As part of the examination, the attending GP notes, “I found it very difficult to pinpoint history today” and “I’m not exactly sure how to help.”

Katheleen was originally due to go on a list for surgery [details removed] in month 4 but this was put off by the hospital due to the “unpredictability” of surgical loads in the winter months. Whilst Kathleen faced the worry [of whether she would have the operation she needed soon enough], she also reported that a consultant had “said ‘[details removed] with all its dangers,’ so I believe there’s some danger in what he’s going to do, but there’s danger in everything isn’t there?” (diary entry, month 15).

### Pamela

During month 8, Pamela attended surgery seemingly at the instigation of her son who felt that she was not taking her medicines as prescribed. The attending GP recorded the suspicion that Pamela was “cognitively impaired” and the consultation led the GP to the view that she was “possibly concealing her symptoms.” On the son’s insistence, Pamela showed the GP [details removed] “which she wasn’t going to show or mention.” The following month, Pamela presented with memory loss for the first time. An interview with her GP highlighted concerns around whether Pamela was taking her prescribed medicines in light of these memory changes, although a dosette box was being use to facilitate compliance.

### Deborah

Deborah’s main problems involved rheumatoid and osteo-arthritis, although when describing her symptoms at initial interview she said that “everyday [brings] something different.” She lived with her husband who was doing “whatever is needed” domestically. Arthritis had a significant impact on multiple joints. At initial interview she spoke of a bone in her leg which was “wearing away” and “there’s not a lot they can do.” This view was confirmed during a GP interview, “That’s why she comes in all the time … we can’t help her properly, we can’t cure the problem.” This situation had led her to consult a range of alternative practitioners, including a faith healer who “didn’t do any good.” Deborah further reported that “something had ruptured” in her shoulder but complained that she had not been sent for a scan. In addition to three other health problems, she also reported recent “memory changes” to the interviewer, about which she had “not spoken to anyone” and hoped “it’s not Alzheimer’s.” Deborah was being seen in secondary care for [details removed].

The main safety issue for Deborah concerned the medicines she was taking, which, as her GP put it during an annual interview, could cause “stomach bleeding or kidney problems … that’s why she’s having these blood tests every month.” The root of the safety risks for this participant appeared to be the additional workload created for both patient and primary care physician in relation to the management and monitoring of specialised [details removed] medication which was not licensed for use in primary care. There is an unfortunate irony that these risks were created against a background where none of the specialist medication was perceived to be having much of an effect.

This clinical background led Deborah’s GP to conceive of her as a “demanding” patient: “So it seems like the woman is here every week.” But the GP added (in annual interview): “even though she’s been here only 4 or 5 times in the winter.” Data gathered for the 24 month period of the study showed she was actually attending once or twice a month. The EHR also revealed some procedural confusion around a prescription for a specialised medication and associated blood monitoring tests.

### Alan

At recruitment, Alan’s main contact with health services consisted of 3 monthly blood tests for diabetes with the practise nurse; a condition which he believed he no longer had. The EHR revealed contradictory information around a statin prescription and a few appointment cancellations.

In month 15, Alan was admitted to hospital by ambulance after collapsing at an event. He put this down to commencing statins two weeks previously. Following further episodes of feeling faint, he stopped taking the statin and mentioned this to his GP at a subsequent appointment, who marks the medical record “not for further lipid modifying medication.” This decision was confirmed by doctor and patient at a subsequent consultation 4 weeks later. In month 19, another prescription was stopped, which, unlike statins does list dizziness and fainting as potential side-effects.

Of all the cases, Alan appeared least engaged with his health problems, although the work of managing his conditions – whether through eating a healthy diet – or organizing the medications he was taking daily, seemed to fall largely to his wife. However, his symptomology (such as in respect of pain) seemed far less than in the other cases on an everyday basis. When asked what all of his tablets were for during his initial interview, he said, “I don’t know you’d have to ask a doctor.”

### Helen

Helen had a large number of active health conditions and was the biggest user of medications in the case subset. At recruitment she reported, among other conditions, a history of asthma, high blood pressure, diabetes, and heart problems. She described herself as “prone to DVTs,” and had previously had a stroke.

In Helen’s case, a large number of “main symptoms” and health conditions were identified by both patient and doctor (including hypertension, asthma, and diabetes), although at baseline the patient did not mention CKD, [details of three medical conditions removed] or chest pain. During the course of the study, Helen was also seen in [four hospital departments and one community service – details removed]. She presented at A&E during months 4 (with chest pain) and 14 [details removed]. In month 6, paramedics attend her house concerning [details removed].

By month 12, a more focused trio of symptoms had emerged in the general panoply of ill health, including an ulcer. In the EHR, reference to a lymphoedema first appears at a consultation in month 12. In months 16 and 17 there are face-to-face consultations about her swollen legs. By month 20 (in a long consultation observed by a researcher), the problem has worsened (appearing as “possible DVT” in the EHR) and a referral (with some access problems) is eventually made to an ambulatory care unit. When asked in interview, the GP said, “We don’t have an answer,” following the investigations on her leg, presenting a “diagnostic dilemma” and “no solution”. In remarking that the patient “likes answers, so does [her partner],” however, the GP appears to focus the problematics of this state of affairs onto the patient and partner experiencing the “anxiety that comes with not knowing what’s going on.”

In safety terms, prescribed medicines were an ever present risk. Helen was previously taken off [a medication which had adverse effects]. She was taking [prescription drug name removed], which can interact with other medications which has to be accounted for in prescribing. In discussing potential safety concerns around Helen’s care, her GP referred to a high chance of her going into chronic kidney failure if she was medicated with diuretics. If she were to use Ace Inhibitors for her CKD, blood tests would need to be undertaken every 2 or 3 days to assess kidney functioning. At the end of the day, there was a danger of her “ending up in hospital.”

### Victoria

Victoria appeared as a comparatively low user of primary care services with only one episode of hospital care in 24 months (outpatients). At recruitment she reported three health conditions including heart failure. The EHR notes “moderate” diverticular disease which the patient discusses, but not with this precise diagnostic label. Only two potential safety issues were evident. Firstly, the GP mentioned that aspirin had not been requested from the patient for a period of time, although it was suggested that Victoria may have been buying it over the counter. Secondly, the GP referred to an incident involving an infection where it was suggested that “rather than waiting until you need to go to A&E,” she could have approached primary care sooner for an antibiotic prescription; thereby avoiding a hospital admission.

## Results III – Thematic analysis

### Instances of potential contributions to patient safety failures

Characteristics of participating patients at baseline are detailed in Table 1. We found examples of most potential contributors to patient safety failures according to the framework described in the study protocol [15]. For access breakdowns [47], all contributory factors from the taxonomy of events were seen. In the case of communication breakdowns [45], all issues were encountered except for the patient side factors “Inarticulateness” [47] and “lack of confidence” [47] and the staff side factor “failure to respond to adverse drug reactions or painful symptoms” [46]. However, we found additional factors that did not seem to fit the taxonomy: “GP doesn’t interact with patient but focuses on computer screen,” “health professional approach seen as patronising” and “health professional has ‘bad attitude.’” So far as errors of coordination and management continuity [46] were concerned, all factors were found except for the patient contributory factor “comprehension errors” [47] and the staff/system error “wrong chart used for patient” [45]. We found additional factors here, mainly related to the intersection between pressures on GP workload, problems arising from seeing different GPs and the ways in which systematised annual review appointments can lack patient-centredness. In the case of relationship breakdowns [45], there was a very good fit between the taxonomy and the data we gathered, with only the patient contributory factor “selfishness” [47] absent. This descriptive analysis highlights how, in a relatively small sample of patients followed over 2 years, contributors to patient safety failures were both frequent and very varied.

Subsequently, we analysed the data extracted via the framework as a whole and in thematic fashion. As well as those themes that derived from our framework (e.g. access, communication), we encountered additional themes, such as “over or under consultation” and “safety problems inherent in health conditions or treatments.” The broad findings coalesced around three main factors, with “work” (for patients) or “workload” (for health workers) as underlying issues: relational tensions in primary care consultations, system constraints in the organisation of care and issues in care continuity.

### Relational tensions and ‘under’ or ‘over’ consultation

Pressure on GPs’ time, and difficulties in securing timely (and lengthy enough) appointments appeared to create anxieties for patients and doctors alike. Some patients avoided seeking help, perhaps believing that their problems were not serious enough (“under consulters”). Other patients, perhaps understood by GPs as being over anxious about their health (“over consulters”), sought repeated appointments, as their complex needs for care and/or emotional support could not be met in a brief appointment slot:

“… if we don’t make … regular appointments then they start ringing in and they’ll see perhaps a locum doctor and then another chest x-ray will be done, you know, when one was done three months ago and another…so …and that’s where continuity comes in, you know. … I think that’s where sometimes reviewing patients who are very anxious about their health, on a pre-planned basis, can be helpful because it saves them sort of seeking multiple … people and getting different opinions, which actually sometimes really worsens her anxiety.” (Irene, GP interview, year 2)

When people brought wider concerns from their lives into the consultation room, this could create additional work for GPs, who are “sitting behind a desk rattling through tons and tons and tons of patients in your surgery”:

“I find it difficult to know what her priorities are … I think that she verbalises problems with her brother, … whether it’s her brother’s health or their relationship. … So I think she takes on a lot of his problems as well. And then puts a lot of her problems to one side. She’s quite a busy person. … That’s her personality and then she becomes busy in the consultation, and it’s difficult to control.” ([Anonymised], GP interview, year 2)

Prioritisation of conditions is well recognised in multimorbidity [51] However, the above extract appears to point to a tension, or disconnect, between organisational or system “needs” (10 min appointment slots, prioritisation) and what a patient needs, wants or expects from the consultation.

An elderly woman was understood to be “coping well at home” despite having heart failure. When she died during the course of the study, her death was seen as “unexpected” and her case was referred to the Coroner’s Office. But the fact that she did not consult meant that she was not on the practice “radar” and was on reflection seen as an *under consulter*:

“She doesn’t consult at all. … She consulted with [GP], he saw her at home to, sort of; transfer the care of her heart failure to Primary Care. … [Another GP] arranged with her to see her every three months and to have her blood checked. Then we have not seen her, there was a telephone consultation with [a different GP] because her bloods showed that she was slightly low on folic acid and [a different GP] just talked to her about her diet and gave her some vitamins and then she passed away.

“She was somebody who from our point of view seemed to, despite her heart failure, coping well at home. Although despite her age and her heart failure perhaps an unexpected death, so I think she was referred to the Coroner’s Officer, so not on our radar really because she wasn't consulting.” (Martha, GP interview, year 2)

In an earlier interview with Martha, it was noteworthy that from the patient’s viewpoint one reason why she was consulting infrequently was that she found it difficult to attend the surgery. Furthermore, she had received a letter from the practice regarding a blood pressure reading, which she did not believe had been taken:

“I don’t go to the surgery, they’re supposed to come here, a nurse came to take my blood pressure, two days later got a letter from [my GP] saying, ‘Pleased your blood pressure has gone down.’ Got another letter two weeks later, saying the same thing, but I hadn’t had another test. Then I got another letter from [my GP] saying pleased and she would take your blood pressure next time you come in. I doubt I could get in, would have to take a taxi, don’t think I’d manage the stairs.” (Martha, health care diary, year 1)

### System constraints

Whilst some threats to patient safety follow from the knowledge, behaviour and inter-personal aspects of human actors, others rather follow from protocols, policies and the ways that systems are organised to provide and deliver care [13]. For example, appointments are difficult to organise; especially when arranged at the last minute. Many GPs work part-time and the increasing use of locum GPs can be seen to have an adverse impact on continuity of care [52]. One GP participant pointed to the value in seeing the same practitioner:

GP: It's far easier to judge the progress of a condition and how the patient's coping with a condition if the same person sees them every time. Sometimes a fresh pair of eyes is good. But mostly having the same doctor can help move things along. I suppose, as I mentioned before, having a clear plan of actions, with timescales. And after that timescale, decision points when it's decided what to do next, is what we're doing working, or if it's not, do we need secondary care? (Larry, GP interview, year 2)

Under these circumstances, the onus can fall on the patient to instigate their own clinical monitoring, rather than relying on system generated recall:

“We rely on her to come back for the blood tests, so if you're not really careful and there's a bigger practice and many doctors or locums come in, probably sometimes you’ll not realise that she hasn’t been for two months for the blood tests, prescribe the next month, and then any side effect or danger in protein loss or the kidneys would then go unnoticed till it's probably further advanced.” (Deborah, GP interview, year 1)

Another case pointed to a failure to undertake a repeat of an “abnormal” blood test, although it was unclear whether this was a “systems” issue, an oversight by an individual member of staff, confusion over whose role it was to recall the patient, or some combination of all of these factors:

GP: …from the blood tests in February, we have advised that it should be repeated in three months and she has not re-attended.

Interviewer: Would she have been sent a reminder?

GP: She hasn’t been sent a reminder, actually, and given that she attended with issues about her memory that is a failing on our part, isn’t it. … [Later] … And so, perhaps both me and our administrator had not picked up on the fact that her blood tests are due. … [Later] … they have not flagged it, we have got a template that flags it up so that they are pulled up by an administrator. … So, there is a bit of a systems failure going on there. ([Anonymised], GP interview, year 2)

During the observation of one consultation, an interaction concerning the results of a laboratory test appeared to point to a presumption of failure in the transfer of test results, with the GP suggesting that attempting to locate the test result was not worth the work involved:

Patient: How did my urine sample go on?

GP: Never got it, so I’m really unsure of what’s happened. As you said, you were better, I didn’t see any point in chasing this up with the lab, because they’re just going to say I can’t find it, is that all right? ([Anonymised], observation of consultation, year 2)

This appears as an example of navigating the challenge between maximising the clinical utility of a test whilst minimising the potential for further workload and treatment burden (i.e. redoing a test that may be unnecessary).

### Issues in work or workload associated with care continuity

Below, a GP reports about a participating patient’s recent referral for expert opinion:

GP: … they saw her that day, which is great. I had no correspondence for that. She phoned me a week later and they said they will do a scan and I’ve not heard, we didn’t receive any correspondence. So … so I phoned up [the] unit and said, ‘This lady has had a scan, this was an admission avoidance, when is the scan going to be?’ They go, ‘We don’t know, we don’t have the records.’ So then I had to phone back a week later, they said they’d look into it, then she phoned back saying, ‘I’ve still not heard,’ so there’s this yoyo that was going around for the best part of two or three weeks, her [removed] were no worse in that time, I did have to see her that once just to reassure myself that nothing had changed. ([Anonymised], GP interview, year 2)

The nature of this scenario creates work and increased workload for the patient, her carers (including health service staff) and suggests “ambiguity and workarounds” [53] between care sectors. Anxiety around clinical decision making in people with multimorbidity or other complexities entails work, and systems constraints exacerbate the work or effort required to resolve the clinical uncertainty.

Another issue related to care transitions was that some specialist medication is not licensed for use in general practice. This was seen to create work for a patient who found it difficult to attend a hospital for screening appointments. This appeared as a potential safety concern:

“… I think the patient probably finds it more difficult to go back to the hospital for the check-ups, which probably in the beginning might be sometimes weekly, so it's a lot of hassle for her but we can't help her there really because it's the licencing and we don’t know… And it is a lot of workload involved there, so we sort of… general practice shies away from that a little bit. … [Later] … Again, if some of those tablets, let’s say, causes stomach bleeding and she's got severe indigestion and thinks, oh, I don’t want to go to the doctor again, and then suddenly a vessel bursts in the stomach and then she's got real problems.” (Deborah, GP interview, year 1)

Informal carers, typically patients’ family members commonly acted as advocates in health service consultations as well as helping out with aspects of home care and management of patient’s conditions at home. However, on occasion they were not willing to take on certain tasks expected of them by the formal health service. For example, whilst Michael’s’s partner had been trained to give a glucose injection should he suffer a diabetic hypo, Helen’s partner was not happy to take on what he saw as a strictly medical role. The work required in managing chronic conditions is again highlighted, here with the added consideration of determining who has the capacity to fulfil the required functions related to the management of care.

In the particular context of workload, one GP noted—and seemingly not ironically—that patients who “under consult” are preferable from a workload viewpoint:

Interviewer: And what is it like, having a patient like [removed], whom you don’t see very often?

GP: It’s great, because you don’t see them, so they don’t cause you any workload, if I’m being absolutely bloody honest with you. I think it’s unsettling when you come to look at their records, and you think, goodness, she has these [removed] conditions and she’s not needed to see us. ([Anonymised], GP interview, year 1)

This patient is seen as “great” because they “don’t cause you any workload” and attend “appropriately” “for monitoring.” Although questions are raised around tensions between managing workload whilst maintaining effective and safe care.

## Results IV – Synthesis of research findings

Overall, the findings pointed to various areas of tension impacting on safety. The first of these was around managing underlying anxieties and uncertainties in interactions, both from a patient and a professional perspective. For example, and as expanded in the discussion (see below), there was evidence of a clash between patient expectations to “do something” (as in repeating investigations) and, from the clinical perspective, the risk of that “something” leading to worse outcomes or even harm.

From a patient safety perspective, managing this inherent tension appears as a potentially key element of clinical practice. That requires more work for clinicians, and even more work when dealing with multimorbidity. Handling this tension can lead to real or perceived ‘over’ consultation – (as exemplified by Irene, cited above). More investigations generate more results which leads to a need for further consultations.

Borderline test results may generate further anxieties, in a kind of negative feedback loop to where we began. This assertion, whilst speculative, brings is to the second area of tension, which involves maximising clinical utility whilst minimising treatment burden. To the ethnographic observer, Helen appeared as someone whose full-time job was organising and attending health appointments. And none of this appeared to address her symptoms or the questions she had about her various conditions. A non-clinician might wonder whether all of this contact with the health service was making her better or worse, with some medications apparently necessary to keep her alive and others which appeared to present the risk of causing her to end up in hospital in their own right.

Systems of care are not necessarily designed to account and effectively reduce the work required to deal with these tensions, and by extension––make things safer. Whether it is possible to effectively deal with the work required to address these tensions in a time bounded routine consultation (and a resource constrained health system) is unclear. Further, our findings show how the current organisation of medical care might exacerbate these constraints. The atypical example in the data of ‘under’ consultation, illuminated a system run so as to reduce the sense of demand and clinician workload, but this can be at the expense of necessary care. GP participant accounts pointed to a sense of relief in less workload, but with an accompanying realisation that the patient’s needs were not necessarily met. Could this be a potential precursor of clinician burn out?

## Discussion

Patient safety work involves further burdens on top of existing workload for both clinicians and patients. In this conceptualisation, safety work seemingly forms a negative feedback loop with patient safety itself. A line of argument drawn from the triangulation of findings from different sources, points to a tension between the desirability of a minimally disruptive medicine versus safety risks associated with the way that services are set up, organised and delivered.

### What is the potential agency of doctors versus patients in ameliorating clinical iatrogenesis against a background of clinical uncertainty?

By incorporating medical records data and an approach focussed on the detail of purposefully selected cases, we had hoped to reveal discrete safety incidents which we might then link back to precursor events. However, on the rare occasions on which we found an observable and identifiable “safety incident,” such as Alan fainting, it is not clear how that might have linked to other safety concerns which had arisen in his case, such as possible antipathy about his state of health, denial of medical diagnosis or cancelling medical appointments. And none of these concerns explain why he linked this incident to a statin prescription; nor why the GP was happy to go along with his view. Although this may have been to appease the patient. Later, another medication was stopped. Perhaps that was the culprit? This example does however underline the reactive nature of the medical system in general terms; and uncertainties around ascribing causes to adverse outcomes, even with the benefit of hindsight. The patient has agency here, and is another factor to be weighed up against clinical guidelines or recommended treatments. Regular diabetes monitoring can prevent serious complications, but Alan believed he did not have the condition; which he felt was temporarily brought on through over consumption of a popular sugary drink when it was on special offer. “Fainting” could be a symptom of diabetes, and if Alan had attended these check-ups more regularly, perhaps he could have avoided blacking out?

For the majority of patients who were more engaged with medicine (even to the point of alleged overuse where anxiety was a compounding clinical feature), there appeared a tension between patient expectations that their GP will “do something,” versus GP concerns that “something else” could lead to harm (e.g. kidney injury) or risk hospital admission with the attendant risks. And thus the nub of the matter appears to be a palpable tension for GPs between meeting patient expectations (via patient-centred care) and preventing adverse events. For patients, feeling safe appears to incorporate elements of feeling satisfied with the quality of the clinical relationship and care, which are intrinsically linked to their expectations and previous experience of services [16]. An essential problem here appears to be that patients and GPs are using different conceptualisations of safety (or satisfaction) which might play off against each other. Interesting questions that emerge from this line of argument are firstly whether patients really expect the kinds of “over treatment” that could increase the risk of adverse events that we have implied from our interpretation of the study data? Secondly, how do GPs manage the tightrope walk between meeting patient expectations to “do something” (which could constitute over treatment and investigation) and “doing no harm,” as in offsetting the potential risks of adding more medicines or repeating investigations?

### In a time and resource constrained system, could a focus on risk management be compromising patient safety?

Having considered some cases in detail, one gets the sense of an ever present backdrop of risks, appearing largely as tasks for GPs as in “avoid hospital admission,” “monitor kidney functioning,” or perhaps “be careful about what you prescribe.” There are different risks for patients perhaps, e.g. of not getting a diagnosis, of the treatment not working or of the GP overlooking or mis-ascribing something. Plus the risk of not getting a sympathetic hearing, the risk of not being taken seriously or the risk of being blamed for the problem or failure to treat it.

The sense then is that both GP and patient have to be on constant alert to a plethora of risks but that when something does go wrong the reasons for it may be uncertain or difficult to pin down. “Let’s stop this drug and see if that helps,” perhaps. Within a time constrained and reactive system, however, the context of multimorbidity can mean that all the available time is spent on reacting to the latest crisis, whilst avoiding serious harm through clinical iatrogenesis. In this scenario, there is no time left to address basic patient needs (e.g. “Why isn’t this drug working? Where’s my test result?”) and it could be said that both clinician and patient appear to be in perpetual workaround; or dancing to a system generated script.

One feature or narrative concerning contemporary medical systems is “information chaos”: “comprised of various combinations of information overload, information underload, information scatter, information conflict, and erroneous information” [54]. Increased workload, including additional working around “chaotic,” complex and inter-twined systems would be expected to increase the likelihood of an error occurring. Data from Primary Care are sparse although a US study of hospital paediatric nurses found that “being rushed” was associated with both medication errors and “burnout.” [55]

In circumstances where increasing clinical workload is a driver of safety failures, more work in monitoring or preparing for a possible adverse reaction or serious adverse event (on top of a focus on reacting to the latest medical problem) seemingly forms part of a negative feedback loop whereby the situation becomes less safe. The more people engage with medicine, whether willingly or unwillingly, the more necessarily they are put at risk of potential harm. Given especially the problematics of conditions and syndromes which are either hard to treat or difficult to define clinically and where existing drug lists might prevent the prescription of usual treatments.

### The emergence of under or over consultation as a potential driver of patient safety failures highlights a tension between the meta-organisation of health care and the communicative, legalistic or performative aspects of medical consultations in the context of patient centred care

A finding which added to our a priori framework concerning patient safety in primary care highlights the contribution of perceived over or under consulting as having a role in precipitating adverse events. There are highly socialised notions about when it is appropriate to use NHS services [56] and such considerations formed a part of the narrative when participants discussed their own patterns of service use. Avoiding the label of “timewaster” can be a moral imperative for patients [57] and the notion of candidacy is useful in explaining health service utilisation where services are seen as being in short supply [58]. On the other hand, whilst it might suit practitioners if patients don’t over consult, this can lead to problems as identified in our findings. Over or under consultation compounds both the discursive aspects of the consultation (where there may be confusion on either side as to the medical reasons for consulting in the first place) and system demands on when patients are seen (as in periodic reviews for patients with, e.g. diabetes or heart disease, which may be viewed as a waste of time by some – results not shown).

Our main findings point to multimorbidity as a magnifier of tensions in the delivery of health services and quality care in general practice. Within this context, older patients with complex and varied healthcare needs encounter almost all potential contributors to patient safety failure, as they negotiate the tensions and limitations of system demands. For GPs, patients and carers, these tensions create additional work which they can struggle to manage and often find frustrating. When the quality of interactions between doctors and their patients begin to fail, or technical problems arise, this can make a bad situation worse in the context of limited consultation times and the perceived scarce or ‘rationed’ nature of medical resources.

These findings fit with the model of “cumulative complexity” whereby factors “accumulate and interact to complicate patient care” [59]. This model is principally concerned with the balance between the workload created by the demands of care and an individual’s capacity to deal with it [59]. The model has previously found to be relevant for people living with multiple chronic conditions [60]. In the introduction, the tendency for a theoretical and empirical focus on patient workload or burdens was noted and a similar preoccupation is evident in the literature concerning patient “capacity” within a framework that is concerned with enabling patients to help themselves more [61]. In this study, whilst we found both patient and staff or system-side threats to safety, “workload” appeared at least as important a patient safety issue when referring to staff as it did to patients. Whilst another strand of health research has illuminated the ways in which health professionals form a part of patients’ relational networks [62], the general tendency can rather be to separate out issues for health service staff from those facing patients. The findings of this study point to a need to consider the ways in which care work and clinical responsibilities are allocated, and the consequences for safety when “overworked” health care staff and patients can seemingly attempt to shift care roles or tasks onto one another. Despite repeated calls for patient-centred care, findings point to the possible limits of patient-centredness [63] when safety is at stake.

### Implications and future work

A focus on multimorbidity has magnified some of the inherent tensions underpinning everyday interactions in primary health care and the examples of over and under consultation have shone a light on some key system issues that need consideration, particularly when trying to improve the delivery of care for people living with complex health and social care needs. The main learning from the study is very much, how can systems work better for everyone concerned and shift away from a focus on professional (or patient) behaviour. In future work we shall be exploring ways in which patients can take a more active role in reducing harm, such as in knowing how to better communicate with their doctors or having a better knowledge of their own medical records. Such efforts will have to be set against the knowledge that any intervention which is experienced as a burden, or does not fit the patient’s self-prioritised needs, is unlikely to succeed.

In relation to potential areas for improving “safety,” one route would seem to involve patient education around acceptance of medical risk, which can seemingly cause some considerable anxiety, dissatisfaction and increased service utilisation. From the practitioner perspective, as well as potentially decreasing patient anxieties, such interventions could have the added benefit of reducing GP workload, which appears as a more tangible threat to safety. Particular results concerning doctor-patient communication in memory loss further underline the importance of creating a climate and space in which patients feel free to discuss concerns around their health. A broader research agenda appears concerning the extent to which entrenched systems of top-down medical care can ever fit with contemporary idealised notions of patient-centred care.

### Strengths and weaknesses

Traditionally, patient safety research has attempted to explain safety failures by identifying incidents and then historically examining the circumstances that led to failure, as in a study of falls in hospital [64]. We adopted a different tack by following patients over time to discern whether and how adverse events occurred and any precursor, precipitatory or contextual factors. The principal strength of our approach is the longitudinal aspect and the triangulation or comparison of data from different sources. Whilst qualitative research cannot discern frequencies or the probability of events occurring in a given population, it is useful for uncovering context and highlighting areas for further research. The principal weakness of our approach probably concerns observer bias, i.e. the presence of researchers in some consultations may have influenced the behaviour of participating patients or health care workers. Further, people with mental health problems were under represented in the sample and previous research has shown they may be at increased risk of patient safety events [39], e.g. related to polypharmacy or medication adherence.

## Conclusion

Multimorbidity creates work for patients, carers and health professionals. The way that the primary care system is currently set up can lead GPs to feel potentially relieved when workload is minimised. However, a focus on managing workload runs the risk of patient safety failures, e.g. when a patient is not seen much by primary care staff. This is potentially relevant for all patients but appears more so for patients living with complex health and social care needs.

Multimorbidity creates burdens for both patients and their doctors which can be seen to create circumstances in which safety can be threatened. The mechanisms by which differential take up of, or access to, primary care consultations may precipitate different types of medical errors or iatrogenic adverse events requires further investigation. Efforts to improve safety in primary care need to tread carefully when intervening in complex clinical management plans, as those which create extra workload for doctors or their patients may cause unintended consequences.
